# Tip-Jump Response of an Amplitude-Modulated Atomic Force Microscope

**DOI:** 10.3390/s120506666

**Published:** 2012-05-22

**Authors:** Po-Jen Shih

**Affiliations:** Department of Civil and Environmental Engineering, National University of Kaohsiung, No. 700, Kaohsiung University Road, Nanzih District, 81148, Kaohsiung, Taiwan; E-Mail: pjshih@nuk.edu.tw; Tel.: +886-7-591-6592; Fax: +886-7-591-9376

**Keywords:** jump, bistability, snapping, hysteresis, intermittence

## Abstract

The dynamic behaviors of an Atomic Force Microscope are of interest, and variously unpredictable phenomena are experimentally measured. In practical measurements, researchers have proposed many methods for avoiding these uncertainties. However, causes of these phenomena are still hard to demonstrate in simulation. To demonstrate these phenomena, this paper claims the tip-jump motion is a predictable process, and the jumping kinetic energy results in different nonlinear phenomena. It emphasizes the variation in the eigenvalues of an AFM with tip-sample distance. This requirement ensures the phase transformations from one associated with the oscillation mode to one associated with the tip-jump/sample-contact mode. Also, multi-modal analysis was utilized to ensure the modal transformation in varying tip-sample distances. In the presented model, oscillations with various tip-sample distances and with various excitation frequencies and amplitudes were compared. The results reveal that the tip-jump motion separates the oscillation orbit into two regions, and the jumping kinetic energy, comparing with the superficial potential energy, leads the oscillation to be bistable or intermittent. The sample-contact condition associates to bifurcation and chaos. Additionally, the jumping is a strong motion that occurrs before the tip-sample contacts, and this motion signal can replace the sample-contact-signal to avoid destroying the sample.

## 1. Introduction

Dynamic atomic force microscopy (AFM) is widely used in high resolution imaging on a nanometer scale. The most commonly used operating mode of dynamic AFM involves a feedback system of amplitude modulation and exploits the fact that the tip of the microcantilever oscillates with amplitudes of a few tens of nanometers. A hard interaction between tip and sample introduces a strong nonlinearity in the motion of the tip; such nonlinearity includes tip-jump, bistability [1,2], snapping, hysteresis, intermittency [3], period doubling, and bifurcation from periodic to chaotic oscillations [4]. These nonlinear behaviors reduce the accuracy of measurement by AFM and should be avoided in making measurements. Some of the above phenomena have been observed experimentally; however, few mathematical models have been developed to simulate or demonstrate the mechanisms. The reasons are that the models are simplified to a single degree of freedom and the stiffness of the microcantilever in AFM does not vary with the tip-sample distance. Therefore, the continuities of the eigenvalues, displacements, and velocity of the microcantilever cannot be verified at the moment of tip-jump and sample-contact.

This paper claims that the tip-jump is a required condition that results in different nonlinear phenomena, and those can be predicted. The tip-jump is caused by the asymmetric two-wall potential that is determined using Liapunov stability theory [5], and the disequilibrium between the restoring force of the microcantilever and the superficial force results in chaos [6]. The tip-jump has been described with reference to some physical phenomena, such as strange contours, unexpected height shifts, and sudden changes in the apparent resolution of acquired images [7–9]. There is no exhaustive description of jumps and their relationship to snapping, bistability, hysteresis, and intermittency. Some studies have, however, addressed the prevention of jumping by controlling geometric properties or excitation frequencies/amplitudes [10,11].

In addition to the nonlinear phenomena, the superficial force that governs the microcantilever of an AFM yields two significant characteristics. The natural frequency of the microcantilever changes directly with the tip-sample distance [12–14], and its motion includes oscillation, tip-jump, and the sample-contact oscillation. Unfortunately, most presented models are based on a constant eigenvalue and do not capture the rapidly change in eigenvalue before/after a jump, and models that are based on single degree of freedom [15–17] cannot simulate the modal transformation before/after jump or contact. As a result, a multi-modal analysis with eigenvalues that vary with the tip-sample distance is required in AFM simulation.

This investigation involves a multi-modal analysis of AFM microcantilever, in which the natural frequencies vary with the tip-sample distance, to ensure the accuracy of oscillation of AFM microcantilever suffering from superficial forces. The tip-jump mechanism was based on force disequilibrium, and a force-displacement diagram helped explain the tip-beginning and tip-ending positions on the superficial potential force curves. Then the discretization method [18,19] was utilized to separate the superficial potential force curve into several piecewise linear segments. Each piecewise linear segment was related to a particular tip-sample distance, and the microcantilever oscillation could be determine exactly for each segment. Moreover, multi-modal analysis and the associated orthogonality conditions [20,21] ensured the continuities at the positions where these segments met and at the transformation between oscillation and tip-jump/sample-contact. The time-dependent boundary conditions modified from Mindlin [22] were also adopted to solve the superficial potential force at the tip-end and the excitation force at AFN base end. Notably, unlike the author's previous study, this paper elucidated the superficial force effects and the tip-jump effect on the nonlinear phenomena. In this study, oscillations driven at various tip-sample distances and excitation frequencies/amplitudes were compared on phase portraits.

This investigation makes three main contributions. It provides an easy understanding of the tip-jump mechanism by demonstrating the force equilibrium. The method used also elucidates the cause of the zero-eigenvalue and points out the tip begin/end positions of the jumping. The second contribution is that this paper claims the tip-jump motion is a predictable process, and the jumping energy comparing with the superficial potential force energy, results in bistability for large kinetic energy and snapping for low kinetic energy. Furthermore, if the tip contacts the sample, the bistable motion may become hysteresis and the snapping motion may become intermittency or chaos. The third contribution is that this paper proposes the tip-jump motion is a strong signal occurred before the tip contacts the sample, and the detected jumping-signals can replace the traditional sample-contact signal to avoid destroying the sample.

## 2. Mathematical Model of Oscillation of Microcantilever

**Figure 1.**
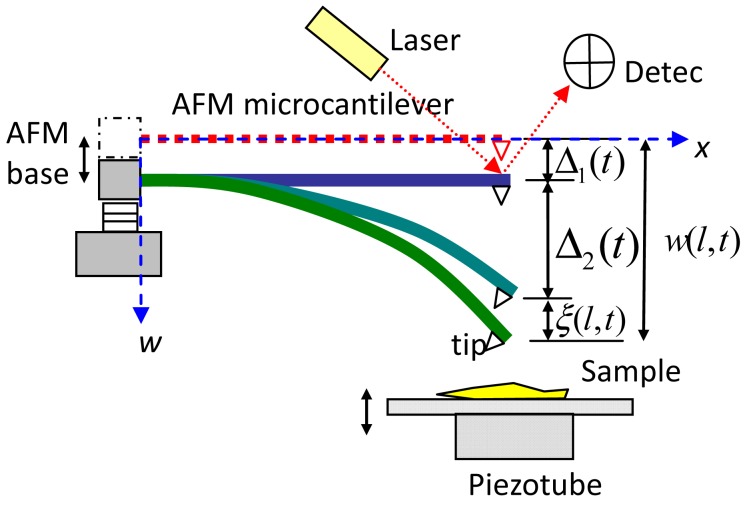
Schematics of the deflection of AFM microcantilever. Δ_1_(t) denotes elevation of AFM base, Δ_2_(*t*) is static deflection caused by deflection dependent tip-end force, and *ξ*(*l, t*) is the dynamic deflection caused by the surface homogeneous wave.

The typical microcantilever in an AFM is constructed from a piezoelectric oscillator at one end with amplitudes that the tip at the free end can tap samples. As shown in Figure 1, the microcantilever was clamped at x = 0 and had a tip at x = L. Table 1 presents the material properties of the soft microcantilever that was made of the si-si 111 material [8]. The microcantilever model was justified because the deflection of the tip-end was small and the thickness of the microcantilevers was less than the length of the beam. The effects of the axial force, transverse shear, and rotary inertia were assumed to be negligible; the tip was assumed to be rigid. The separation of variables was used in this linear elastic analysis. The excitation of AFM base was subjected to be a sinusoidal wave, and the microcantilever motion was obtained exactly in every piecewise linear segment of the superficial potential curve.

### 2.1. Decoupled Equations of Motion

The elastic Bernoulli-Euler equation of the microcantilever motion is:

(1) $$\frac{\partial^{2}}{\partial x^{2}}[EI(x)\frac{\partial^{2}w(x,t)}{\partial x^{2}}]+m(x)\frac{\partial^{2}w(x,t)}{\partial t^{2}}+c\frac{\partial w(x,t)}{\partial t}=q(x)p(t)$$

Where *w*(*x,t*) is the deflection; *E* is Young's modulus; *I*(*x*) is the moment of inertia and is assumed to be constant; *m*(*x*) is the mass per unit length with the microcantilever assumed to be homogeneous); *c* is the damping coefficient, and *q*(*x*)*p*(*t*) represents the applied force per unit length on the microcantilever. The deflection of the microcantilever is modified from the work of Mindlin and Goodman [22]:

(2) $$w(x,t)=\xi (x,t)+\sum_{i=1}^{2}\Delta_{i}(t)g_{i}(x)$$

in which ξ(*x,t*) is the surface homogeneous wave function, and Δ*_i_*(*t*)**g***_i_*(*x*) is the shift-time function associated for time-dependent boundary conditions. Δ*_1_*(*t*)**g***_1_*(*x*) is the rigid body motion along the microcantilever that is caused by AFM base; Δ*_2_*(*t*)**g***_2_*(*x*) represents the static deflection with AFM base at various positions. Substituting Equation (2) into Equation (1) yields:

(3) $$EI\xi^{IV}+m\xi +c\xi =q(x)p(t)-\sum_{i=1}^{2}\left[EI\Delta_{i}g_{i}^{IV}+m{\ddot{\Delta }}_{i}g_{i}+c{\dot{\Delta }}_{i}g_{i}\right]$$

The deflection ξ(*x,t*) under constant boundary conditions is expressed by:

(4) $$\xi (x,t)=\sum_{n=1}^{\infty }\Phi_{n}(x)T_{n}(t)$$

in which **Φ**_n_(*x*) are the mutually orthogonal mode shape functions; and *T_n_*(*t*) are the mode time functions. Orthogonality can be used to expand *g_i_*(*x*), *g_i_^IV^*, and *q_i_*(*x*) in Equation (3) as a series of mode functions:

(5) $$g_{i}(x)=\sum_{n=1}^{\infty }G_{i,n}\Phi_{n}(x)$$

(6) $$g_{i}^{IV}(x)=\sum_{n=1}^{\infty }G_{i,n}^{*}\Phi_{n}(x)$$

(7) $$q_{i}(x)=\sum_{n=1}^{\infty }Q_{n}\Phi_{n}(x)$$

in which the coefficients (*G_i,n_*, $G_{i,n}^{*}$, *Q_n_*) depend on the boundary conditions of the tip-end. Substituting Equations (5)–(7) into Equation (3), dividing both sides by *m***Φ**_n_(*x*)*T_n_*(*t*), and setting the equation equal $\omega_{n}^{2}$ yield the equations:

(8) $$EI\Phi_{n}^{IV}-m\omega_{n}^{2}\Phi_{n}=0$$

(9) $$m{\ddot{T}}_{n}+c{\dot{T}}_{n}+\omega_{n}^{2}mT_{n}=p(t)Q_{n}-\sum_{i=1}^{2}\left[EI\Delta_{i}G_{i,n}^{*}+m{\ddot{\Delta }}_{i}G_{i,n}+c{\dot{\Delta }}_{i}G_{i,n}\right]$$

The solutions to Equations (8) and (9) are:

(10) $$\Phi_{n}=C_{n}\cos\beta_{n}x+D_{n}\sin\beta_{n}x+E_{n}\cosh\beta_{n}x+F_{n}\sinh\beta_{n}x$$

(11) $$T_{n}=e^{-\zeta w_{n}(t-t_{0})}\left(A_{n}(t_{0})\cos[\omega_{Dn}(t-t_{0})]+\frac{B_{n}(t_{0})+A_{n}(t_{0})\zeta \omega_{n}}{\omega_{Dn}}\sin[\omega_{Dn}(t-t_{0})]\right)+\frac{1}{m\omega_{Dn}}\int_{t_{0}}^{t}\left\{e^{-\zeta w_{n}(t-\tau )}\sin\omega_{Dn}(t-\tau )\left(Q_{n}p(t)-\sum_{i=1}^{2}[EI\Delta_{i}G_{n}^{*}+m{\ddot{\Delta }}_{i}G_{n}+c{\dot{\Delta }}_{i}G_{n}\right)\right\}\text{d}\tau$$

where *ζ* = *c*/(2*mω_n_*), $\beta_{n}=\sqrt{\omega_{n}/\sqrt{EI/m}}$, and $\omega_{Dn}=\omega_{n}\sqrt{1-\zeta^{2}}$. The coefficients (*C_n_, D_n_, E_n_, F_n_*) can be obtained by applying boundary conditions at the base-end and the tip-end, and [*A_n_*(*t*_0_), *B_n_*(*t*_0_)] can be obtained by applying the initial conditions in every piecewise linear segment. Note that the integration intervals in Equation (11) should be relatively smaller than the jumping interval when the numerical accuracy is considered.

### 2.2. Analysis of Geometric Modes under Boundary Conditions

The clamped end of the microcantilever (*x* = 0) is supported by the piezoelectric oscillator, and the boundary conditions are:

(12) $$w(0,t)=S(\omega_{f}t)$$

(13) $$w^{\prime }(0,t)=0$$

(14) $$w^{\prime \prime }(l,t)=0$$

in which *S*(*ω_f_t*) is the amplitude of excitation at frequency *ω_f_*. The boundary conditions at the tip-end involve the superficial force *F*(*w*):

(15) $$EIw^{\prime \prime \prime }(l,t)+F(w)=0$$

where *F*(*w*) is defined by the Lennard Jones (LJ) potential force:

(16) $$F(w)=-\frac{A_{1}R}{180{\left(Z-w\right)}^{8}}+\frac{A_{2}R}{6{\left(Z-w\right)}^{2}},$$

in which *Z* is the distance between AFM base and the sample; *R* is the radius of the tip, and *A*_1_ and *A*_2_ are the Hamaker constants. *F*(*w*) is discretized into several piecewise linear segments, *F^i^*(*w*); and accordingly, *F^i^*(*w*) is expanded at with a slope of $k_{i}^{*}$, and $F^{i}(w)=F_{0}^{i}+k_{i}^{*}(w-w_{0})=F_{0}^{i}+k_{i}^{*}\zeta$. Substituting Equation (2) into Equations (12)–(15) yields the boundary conditions of the mode functions:

(17) $$\xi (0,t)+\sum_{i=1}^{2}\Delta_{i}g_{i}(0)=S(\omega_{f}t)$$

(18) $$\xi^{\prime }(0,t)+\sum_{i=1}^{2}\Delta_{i}g_{i}^{\prime }(0)$$

(19) $$\xi^{\prime \prime }(l,t)+\sum_{i=1}^{2}\Delta_{i}g_{i}^{\prime \prime }(l)=0$$

(20) $$EI[\xi^{\prime \prime \prime }(l,t)+\sum_{i=1}^{2}\Delta_{i}g_{i}^{\prime \prime }(l)]+F_{i}(w)=0$$

Let Δ_1_ = *S*(*ω_f_t*) and $\Delta_{2}=F_{0}^{i}l^{3}/(3EI)$. The boundary conditions can be rewritten:

(21) $$\begin{array}{llll} \xi (0,t)=0, & \xi^{\prime }(0,t)=0, & \xi^{\prime \prime }(l,t)=0, & EI\xi^{\prime \prime \prime }(l,t)+k_{i}^{*}\xi =0 \end{array}$$

(22) $$\begin{array}{llll} g_{1}(0)=1, & g_{1}^{\prime }(0)=0, & g_{1}^{\prime \prime }(l)=0, & {g_{1}}^{\prime \;\prime \;\prime }(l)=0 \end{array}$$

(23) $$\begin{array}{llll} g_{2}(0)=0, & g_{2}^{\prime }(0)=0, & g_{2}^{\prime \prime }(l)=0, & EI\Delta_{2}{g_{2}}^{\prime \;\prime \;\prime }(l)+F_{0}^{i}=0 \end{array}$$

The polynomials of *g_1_* and *g_2_* that satisfy Equations (22) and (23) are obtained by:

(24) $$g_{1}(x)=1$$

(25) $$g_{2}(x)=(3lx^{2}-x^{3})/(2l^{3})$$

Applying Equations (4) and (10) to Equation (21) yields *C_n_* = −*E_n_* and *D_n_* = −*F_n_*; the eigenvalues *β*_n_ can be numerically determined by solving:

(26) $$EI\beta_{n}^{3}[1+\cos\beta_{n}l\cosh\beta_{n}l]+k_{i}^{*}[\sin\beta_{n}lcoh\beta_{n}l-\cos\beta_{n}l\sinh\beta_{n}l]=0$$

This equation describes a clamped-free condition when $I\beta_{n}^{3}\gg k_{i}^{*}$, and a clamped-pinned conditions when $EI\beta_{n}^{3}\ll k_{i}^{*}$. The eigenvalue *β*_n_, depending on $k_{i}^{*}$, vary among the above piecewise linear segments. Moreover, the orthogonality conditions in this modal analysis can be modified from the author's earlier study [20]:

(27) $$\int_{0}^{l}m\Phi_{m}(x)\Phi_{n}(x)\text{d}x=0$$

(28) $$\int_{0}^{l}\left[EI\Phi_{m}^{\prime \prime }(x)\Phi_{n}^{\prime \prime }(x)-\delta (x-l)k^{*}\Phi_{m}(x)\Phi_{n}(x)\right]\text{d}x=0$$

### 2.3. Continuities at Interfaces between Adjacent Segments

When the tip of the microcantilever oscillates from one segment to another one, the displacement and velocity conditions in the former segment are regarded as the initial conditions in the new segment. The orthogonality conditions derived in Equations (27) and (28) are used to transform the dynamics from the previous modal basis to the new modal basis. At the interchange time *t*_0_, the initial conditions *w*_0_(*x,t*_0_) and *ẇ*_0_(*x,t*_0_) those were obtained from previous segment are expanded in new modal basis:

(29) $$w_{0}(x,t_{0})=\sum_{n=1}^{\infty }\Phi_{n}(x)T_{n}(t_{0})+\sum_{i=1}^{2}\Delta (t_{0})g_{i}(x)$$

(30) $${\dot{w}}_{0}(x,t_{0})=\sum_{n=1}^{\infty }\Phi_{n}(x){\dot{T}}_{n}(t_{0})+\sum_{i=1}^{2}{\dot{\Delta }}_{i}(t_{0})g(x)$$

Applying Equations (27) and (28) to Equations (29) and (30) yields the time coefficients at the initial time *t*_0_ of the interchange:

(31) $$T_{n}(t_{0})=\int_{0}^{l}[w_{0}(x,t_{0})-\sum_{i=1}^{2}\Delta_{i}(x)g_{i}(t_{0})\Phi_{n}(x)\text{d}x/\int_{0}^{l}\Phi_{n}(x)\Phi_{n}(x)\text{d}x$$

(32) $${\dot{T}}_{n}(t_{0})=\int_{0}^{l}[{\dot{w}}_{0}(x,t_{0})-\sum_{i=1}^{2}\Delta_{i}(x)g_{i}(t_{0})\Phi_{n}(x)\text{d}x/\int_{0}^{l}\Phi_{n}(x)\Phi_{n}(x)\text{d}x$$

### 2.4. Dynamic Solutions under Sinusoidal Oscillation

Equations (8) and (9) have exact solutions when the piezoelectric oscillator of AFM base is driven by a certain sinusoidal amplitude, *S*(*ω_f_t*) = *A_m_*sin(*ω_f_t*), in which *A_m_* is the amplitude and *ω_f_* is the frequency. The applied forces *p*(*t*)*Q_n_* are set zero. Since *g_i_^W^* = 0, $G_{i,n}^{*}=0$, Δ_1_ = *A_m_*sin(*ω_f_t*) yields ${\ddot{\Delta }}_{1}=-A_{m}\omega_{f}^{2}\sin(\omega_{f}t)$, and $\Delta_{2}=F_{0}^{i}l^{3}/(3EI)$ yields Δ̈_2_ = 0. Rather, Equation (9) is simplified by:

(33) $${\ddot{T}}_{n}+2\varsigma \omega_{n}{\dot{T}}_{n}+\omega_{n}^{2}T_{n}=G_{1,n}A_{m}\omega_{f}(w_{f}\sin\omega_{f}t-2\varsigma w_{n}\cos\omega_{f}t)$$

Its solution can be obtained:

(34) $$T_{n}(t)=e^{-\varsigma \omega_{n}(t-t_{0})}[{\bar{A}}_{n}(t_{0})\cos\omega_{Dn}(t-t_{0})+{\bar{B}}_{n}(t_{0})\sin\omega_{Dn}(t-t_{0})]+\left(A_{m}G_{1,n}\omega_{f}^{2}[(1-\beta_{n}^{2})\sin\omega_{f}t-2\varsigma \beta_{n}\cos\omega_{f}t]\right)/\left(\omega_{n}^{2}[{\left(1-\beta_{n}^{2}\right)}^{2}+{\left(2\varsigma \beta_{n}\right)}^{2}]\right)+\left(2\varsigma A_{m}G_{1,n}\omega_{n}\omega_{f}[(1-\beta_{n}^{2})\cos\omega_{f}t+2\varsigma \beta_{n}\sin\omega_{f}t]\right)/\left(\omega_{n}^{2}[{\left(1-\beta_{n}^{2}\right)}^{2}+{\left(2\varsigma \beta_{n}\right)}^{2}]\right)$$

in which:

(35) $${\bar{A}}_{n}=w(t_{0})-\frac{A_{m}G_{1,n}\omega_{f}^{2}[(1-\beta_{n}^{2})\sin\omega_{f}t_{0}-2\varsigma \beta_{n}\cos\omega_{f}t_{0}]}{\omega_{n}^{2}[{\left(1-\beta_{n}^{2}\right)}^{2}+{\left(2\varsigma \beta_{n}\right)}^{2}]}-\frac{2\varsigma A_{m}G_{1,n}\omega_{f}[(1-\beta_{n}^{2})\cos\omega_{f}t_{0}+2\varsigma \beta_{n}\sin\omega_{f}t_{0}]}{\omega_{n}^{2}[{\left(1-\beta_{n}^{2}\right)}^{2}+{\left(2\varsigma \beta_{n}\right)}^{2}]}$$

(36) $${\bar{B}}_{n}=\frac{\dot{w}(t_{0})\varsigma \omega_{n}{\bar{A}}_{n}}{w_{Dn}}-\frac{A_{m}G_{1,n}\omega_{f}^{3}[(1-\beta_{n}^{2})\sin\omega_{f}t_{0}+2\varsigma \beta_{n}\cos\omega_{f}t_{0}]}{\omega_{Dn}\omega_{n}^{2}[{\left(1-\beta_{n}^{2}\right)}^{2}+{\left(2\varsigma \beta_{n}\right)}^{2}]}-\frac{2\varsigma A_{m}G_{1,n}\omega_{n}\omega_{f}^{2}[-(1-\beta_{n}^{2})\cos\omega_{f}t_{0}+2\varsigma \beta_{n}\sin\omega_{f}t_{0}]}{w_{Dn}\omega_{n}^{2}[{\left(1-\beta_{n}^{2}\right)}^{2}+{\left(2\varsigma \beta_{n}\right)}^{2}]}.$$

Applying the orthogonality conditions to *g_i_*(*x*) yields the coefficient functions:

(37) $$G_{i,n}=\int_{0}^{l}g_{i}(x)\Phi_{n}(x)\text{d}x/\int_{0}^{l}\Phi_{n}(x)\Phi_{n}\text{d}x.$$

Coefficients *G_1,n_* and *G_2,n_* can be exactly obtained as:

(38) $$G_{1,n}=2[1-(1+\cos\beta_{n}l\cosh\beta_{n}l)/(\cos\beta_{n}l\cosh\beta_{n}l)]/\left[\beta_{n}\int_{0}^{l}\Phi_{n}(x)\Phi_{n}\text{d}x\right],$$

(39) $$G_{2,n}=\left\{[c_{n}\frac{2l^{3}}{\beta_{n}}-D_{n}(\frac{6}{\beta_{n}^{4}}-\frac{3l^{2}}{\beta_{n}^{2}})]\sin\beta_{n}l+[-C_{n}(\frac{6}{\beta_{n}^{4}}+\frac{3l^{2}}{\beta_{n}^{2}})-D_{n}\frac{2l^{2}}{\beta_{n}}]\cos\beta_{n}l+[-C_{n}\frac{2l^{2}}{\beta_{n}}+D_{n}(\frac{6}{\beta_{n}^{4}}-\frac{3l^{2}}{\beta_{n}^{2}})]\sinh\beta_{n}l+[C_{n}(\frac{6}{\beta_{n}^{4}}-\frac{3l^{2}}{\beta_{n}^{2}})-\frac{2l^{3}}{\beta_{n}}D_{n}]\cosh\beta_{n}l\right\}/\left[2l^{3}{\int_{0}^{l}\Phi_{n}(x)\Phi }_{n}(x)\text{d}x\right],$$

in which the norm satisfies:

(40) $$\int_{0}^{l}[\Phi_{n}(x)\Phi_{n}(x)]\text{d}x=\left[\cosh\beta_{n}l(C_{n}^{2}+D_{n}^{2})\sinh\beta_{n}l-2\sin\beta_{n}l)+\cos\beta_{n}l\times (C_{n}^{2}-D_{n}^{2})(\sin\beta_{n}l-2\sinh\beta_{n}l)+2C_{n}D_{n}{\left(\sin\beta_{n}-\sinh\beta_{n}l\right)}^{2}+2C_{n}^{2}\beta_{n}l\right]/(2\beta_{n})$$

Substituting Equations (34), (38), and (39) into Equations (2), (4), and (5) yields the exact solutions for every segment.

### 2.5. Jump Mechanism

**Figure 2.**
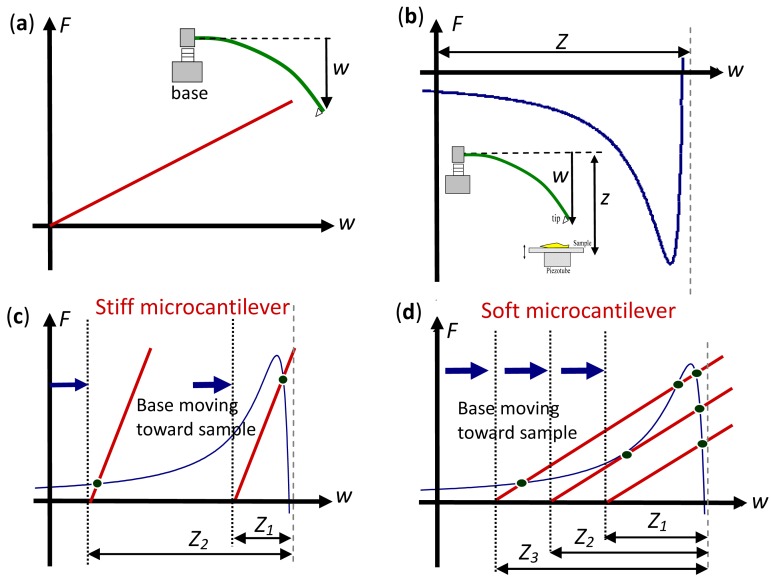
(**a**) Force-displacement relationship for microcantilever. (**b**) Force-displacement relationship for the LJ potential curve. (**c**) For a stiff microcantilever, cases of single cross point are shown. (**d**) For the soft microcantilever, cases of one, two, and three cross points are shown.

Figure 2(a,b) shows force-displacement relations of the restoring force of the microcantilever and the LJ potential force. The LJ potential curve is plotted upside-down and with the restoring force curve of the microcantilever, that is modified from Ashhab *et al.* [23]. The points where these two curves cross represent the equilibrium positions of the tip. Accordingly, the LJ potential curve that is related to a restoring force curve of a stiff microcantilever is compared with that related to a restoring force curve of a soft microcantilever in Figure 2(c,d). The stiff microcantilever is associated with one cross point when AFM base moves toward sample. However, the soft microcantilever case exhibits one, two, or three cross points.

**Figure 3.**
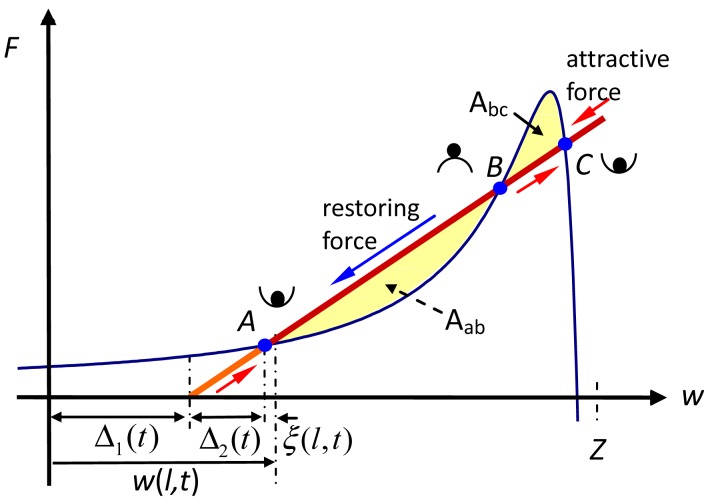
Schematics of the three equilibrium points for soft stiffness microcantilever.

In Figure 3, the cross points *A* and *C* are stable points, and the cross point *B* is a saddle point. When a small *ξ*(*l,t*) disturbs the tip to the right of Point *B*, the restoring force, which exceeds the attracting force, allows the tip to move upward and then to oscillate around Point *A*. When *ξ*(*l,t*) disturbs the tip to the left of Point B, the attracting force causes the tip to move downward and then to oscillate around Point *C*. As a result, the tip of the soft microcantilever oscillates naturally around its equilibrium position. No jump occurs in this free vibration.

**Figure 4.**
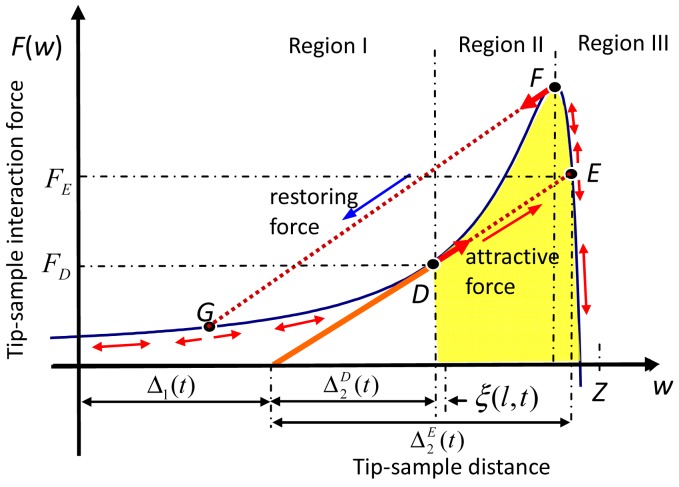
Schematics of tip-jump mechanism. When the microcantilever restoring force curve meets the tangent line at Point *D* (or F), a small disturbance may move the microcantilever from Point *D* (or *F*) to Point *E* (or *G*).

In Figure 4, as the AFM base approached the sample that Δ_1_(*t*) increased, the critical positions were utilized to determine where the tip-jump began and ended. Once, the line of the restoring force became a tangent to the potential curve. Points *A* and *B* in Figure 3 degenerated to Point *D*, and Point *C* in Figure 3 was replaced by Point *E*. Then AFM base continued to move toward the sample, and the unbalanced attractive force allowed the tip to jump from Point *D* to Point *E*. Then the released attractive energy caused the tip oscillate around Point *E* in Region III. Next, when AFM base was retracted, the unbalanced restoring force caused the tip to jump from Points *F* to *G* and then to oscillate in Region I. Figure 4 is easy to be understood since it bases on force equilibrium not on the numerical results when it is compared with other method.

To estimate the displacements and the velocities of the microcantilever after jump occurred, a model was developed by using the conservation of energy. The tip-jump from Point *D* to Point *E* was considered as an example, and that from Point *F* to Point *G* was similar. The tip-jump was assumed to occurred immediately after leaving Point *D* or *F*, and the tip-jump concerned only on the static attractive deflection term $\Delta_{2}^{E}(t)$, the concept was modified from [24]. Before jump, the velocity of the microcantilever is:

(41) $${\dot{w}}^{D}(x)={\dot{\xi }}_{0}(x,t)+{\dot{\Delta }}_{1}(t)g_{1}(x).$$

After jump, the velocity of the microcantilever is:

(42) $${\dot{w}}^{E}(x)={\dot{\xi }}_{0}(x,t)+{\dot{\Delta }}_{1}(t)g_{1}(x)+{\dot{\Delta }}_{2}^{E}(t)g_{2}(x).$$

Consider the variation of the kinetic energy of the microcantilever:

(43) $$\Delta E_{k}=\int_{0}^{l}\frac{m{\left({\dot{w}}^{E}\right)}^{2}-m{\left({\dot{w}}^{D}\right)}^{2}}{2}\text{d}x=\int_{0}^{l}\frac{m}{2}\{{\left({\dot{\Delta }}_{2}^{E}\right)}^{2}g_{2}^{2}+2g_{2}{\dot{\Delta }}_{2}^{E}({\dot{\xi }}_{0}+{\dot{\Delta }}_{1}g_{1})\}\text{d}x.$$

The total potential energy between Points *D* and *E* is:

(44) $$\Delta U=\int_{W_{D}}^{W_{E}}F(w)\text{d}w-\frac{\left(w_{E}-w_{D})(F_{E}+F_{D}\right)}{2}.$$

Finally, the total potential energy equals the kinetic energy Δ*E_k_* = Δ*U*. The unknown ${\dot{\Delta }}_{2}^{E}$, could be obtained numerically. Accordingly, *w^E^*(*x*) and *ẇ^E^*(*x*) are the initial conditions for the next segment of oscillation in Region III.

## 3. Numerical Evaluation and Discussion

### 3.1. Variations of Eigenvalues and Mode Shape Functions

**Figure 5.**
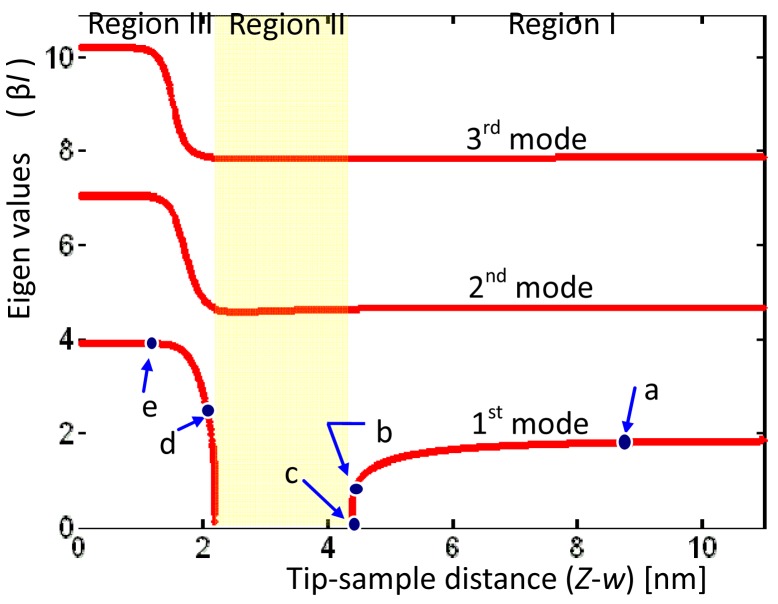
Relationship between first three eigenvalues and tip-sample distance.

**Figure 6.**
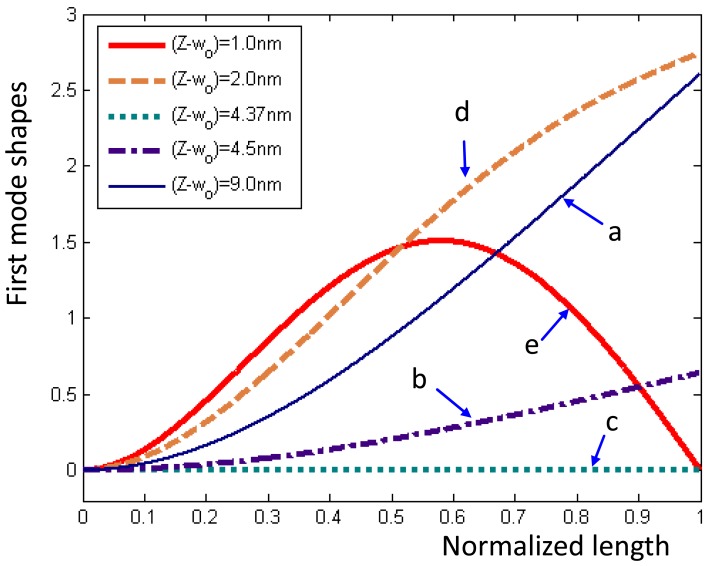
First mode shape functions for various tip-sample distances in Figure 5.

The eigenvalues of the microcantilever with respect to the tip-sample distance were obtained numerically by using Equation (26). Figure 5 displays the first eigenvalues sharply decreasing from right to left in Region I and remaining at zero in Region II. Then the first eigenvalue rises from right to left and then remains constant in Region III. The region in which first eigenvalue is zero is the area of disequilibrium where the jump occurs. The mode shape functions were obtained using Equation (10). Figure 6 plots the first mode shape functions for five tip-sample distances (Z-w), denoted ‘a, b,…,d’, corresponding to the positions ‘a, b,…,d’ in Figure 5. As (Z-w) decreases, the mode shape function changes from a cantilever-type ‘a,’ through an unbalance ‘c,’ to a clamped-pinned beam-type ‘d.’ The above result indicates a significant characteristic of AFM.

### 3.2. Vibration with Initial Tip Disturbance

When the AFM base was located at a particular elevation, the microcantilever bent and the tip moved to the equilibrium point (Point A or C in Figure 3). Once the microcantilever underwent a minor disturbance, a free vibration occurred and the tip oscillated around its equilibrium point until the energy was dissipated.

**Figure 7.**
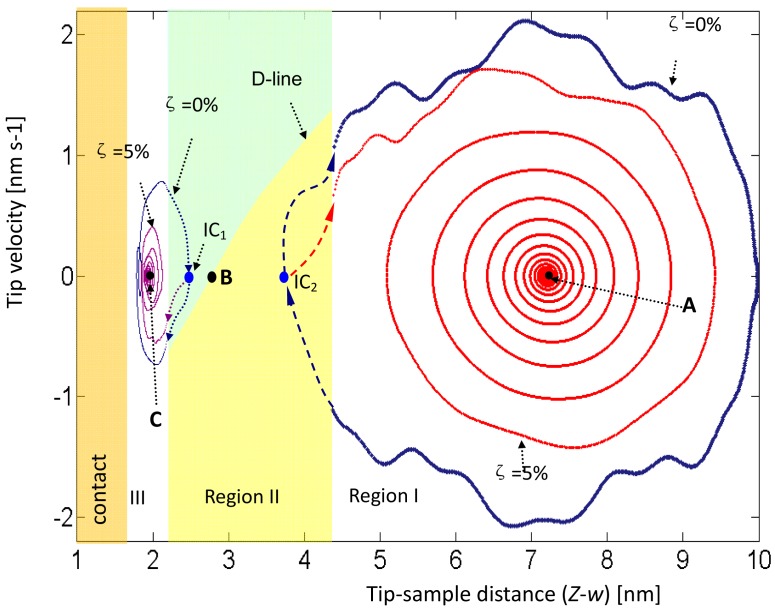
Phase portrait of tip under free vibration with two tip initial conditions and various damping ratios.

In the following numerical discussion, 30 piecewise linear segments were considered within a certain region (11 nm) from the sample to AFM base. Note that the interval in each piecewise segment is setup to be around 1/3 times small than the jumping interval to ensure the simulation accuracy. In this case, the base was arbitrarily set up at Δ_1_ = 8.084 nm, and the equilibrium at Points A and C were obtained at 7.2 and 1.935 nm. Figure 7 presents phase portraits of free vibrations under various initial conditions. The numerical analysis considered the first three modes. IC_1_ or IC_2_ represents an initial disturbance of displacement and velocity of the tip. In these two cases, motion begins in Region II, and the tip travels into Region I or Region III, oscillates around the equilibrium points.

A demarcation line (D-line) in Region II is introduced to distinguish the oscillation vibration around Point A from that around Point C. When the tip is initially located above the D-line, the tip finally oscillates around Point C; otherwise, the tip finally oscillates around Point A. The D-line can be obtained by finding the points whose potential energy equal to the potential energy at Point B, where Point B is the cross-points as discussed in Figure 3. The two 0%-damped orbits show the oscillations that included jumps and periodically circled around Point A and Point C. The two 5%-damped orbits examples of dissipation orbits and finally sink to Points A and C.

Unlike damped driven spring systems that have been described elsewhere, this system exhibited a jump region that separated AFM dynamics into two oscillation systems. One oscillation system was in Region I and exhibited noncontact oscillation, and its behavior was simple. The other oscillation system was in Region III and involved noncontact and contact states; the oscillating behavior in contact state was complicated and was the most likely cause of bifurcation and chaos.

### 3.3. Forced Vibration with Various Driven Amplitudes

**Figure 8.**
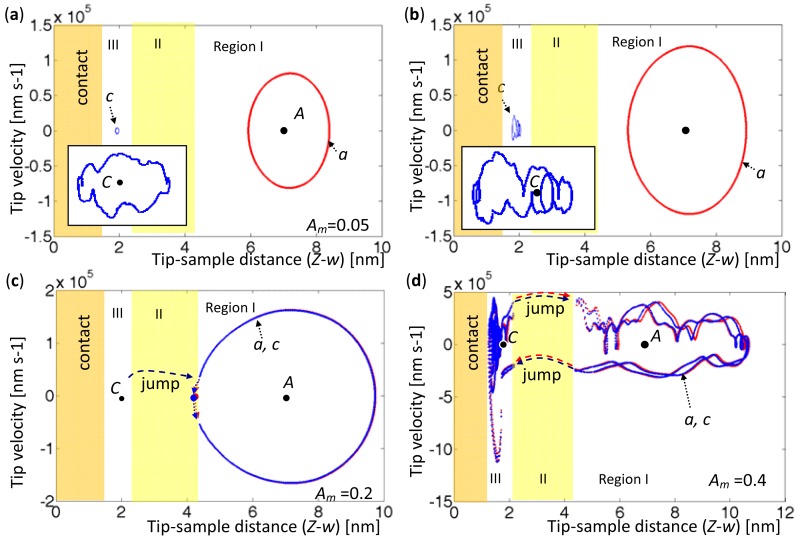
Tip phase portraits with various driving amplitudes at $\omega_{f}^{A}=6.5575\times 10^{4}\text{rad}{\text{s}}^{-1}$. The orbits marked by ‘*a*’ (red) are driven at Point *A* (*Z-w* = 7.2 nm), and the orbits marked by ‘*c*’ (blue) are driven at Point *C* (*Z-w* = 1.935 nm) (**a**) *A_m_* = 0.05 nm. (**b**) *A_m_* = 0.09 nm. (**c**) *A_m_* = 0.2 nm. (**d**) *A_m_* = 0.4 nm.

**Figure 9.**
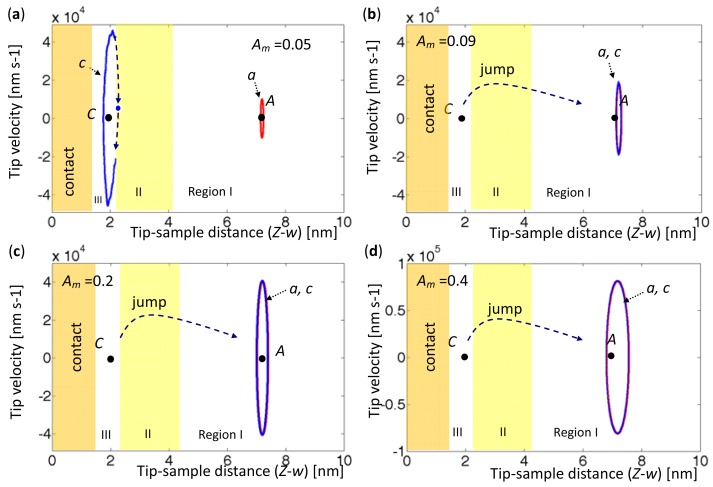
Phase portraits with various driving amplitudes at $\omega_{f}^{C}=2.12447\times 10^{5}\text{rad}{\text{s}}^{-1}$. The orbits marked by ‘*a*’ (red) are driven at Point *A* (*Z-w* = 7.2 nm), and the orbits marked by ‘*c*’ (blue) are driven at Point *C* (*Z*-w = 1.935 nm). (**a**) A_m_ = 0.05 nm. (**b**) *A_m_* = 0.09 nm. (**c**) *A_m_* = 0.2 nm. (**d**) *A_m_* = 0.4 nm.

When the AFM base was set at a certain height elevation, two forced vibrations were driven at two equilibrium points with various amplitudes. In the cases considered herein, area A_ab_ was set to exceed area A_bc_, as shown in Figure 3, since the tip always approached the sample from far away. Accordingly, the oscillation around A was more stable than that oscillation around C. Figures 8 and 9 the tip phase portraits under sinusoidal forced excitation with various amplitudes, *A_m_* = 0.05, 0.09, 0.2, and 0.4 nm. The first 20 modes were considered in the simulation. Two excitation frequencies were set to values determined by the first eigenvalues when the tip was located at Points *A* (*Z-w* = 7.2 nm) and Point *C* (*Z-w* = 1.935 nm). They were $\omega_{f}^{A}=6.5575\times 10^{4}\text{rad}{\text{s}}^{-1}$ in Figure 8 and $\omega_{f}^{C}=2.12447\times 10^{5}\text{rad}{\text{s}}^{-1}$ in Figure 9. The orbit *a*'s (red) and the orbit *c*'s (blue) represent oscillations triggered from *A* and *C*, respectively; the steady state orbits were acquired to demonstrate the final periodicity.

Figure 8(a,b) presents the phase portraits around the equilibrium points with amplitudes *A_m_* = 0.05 and 0.09 nm. Period doubling is observed in the orbits marked by ‘*c*’, and the number of period doublings seems to depend on the amplitudes of oscillation and the sample-contact condition. In Figure 8(c) with *A_m_* = 0.2 nm, the orbit *c* initially circles around *C*; then snaps to Region I, and finally circles around *A*. The tip does not return to Region III because its amplitude is too low to leave in Region I. In Figure 8(d), with the excitation amplitude *A_m_* = 0.4 nm, both orbits oscillate through three regions and exhibit hysteresis, and 14 period doublings occur. Unlike a damped driven spring system, which exhibits contact and noncontact behaviors, AFM oscillates in Region I, jumps in Region II, and exhibits either noncontact or contact oscillation in Region III. The results also indicate bistability when the amplitude is sufficiently large, period doubling when the vibrating tip is shown in Region III, and snapping when the amplitude is too small to exhibit hysteresis.

The excitation frequency was set to $\omega_{f}^{C}$ in Figure 9, where $\omega_{f}^{C}$ was the natural frequency when the tip was located at Point *C*. The excitation amplitude was set to *A_m_* = 0.05, 0.09, 0.2, and 0.4 nm in Figure 9(a–d), respectively. In Figure 9(a), the amplitude of oscillation of the orbit *a* (red) is less than that in Figure 8(a), but that of the orbit *c* (blue) is greater. In Figure 9(b–d), applying the same excitation frequency but different excitation amplitudes yields the orbit *c* that initially oscillates in Region III; jumps to Region I, and then remains in Region I, oscillating with small amplitude. The orbits marked by ‘*a*’ remain stable in Region I. Hence, the excitation frequency $\omega_{f}^{C}$, obtained from Region III, affects large oscillation amplitude in Region III, causing it to snap into Region I. The excitation frequency $\omega_{f}^{C}$ produces extremely stable oscillation in Region I.

**Figure 10.**
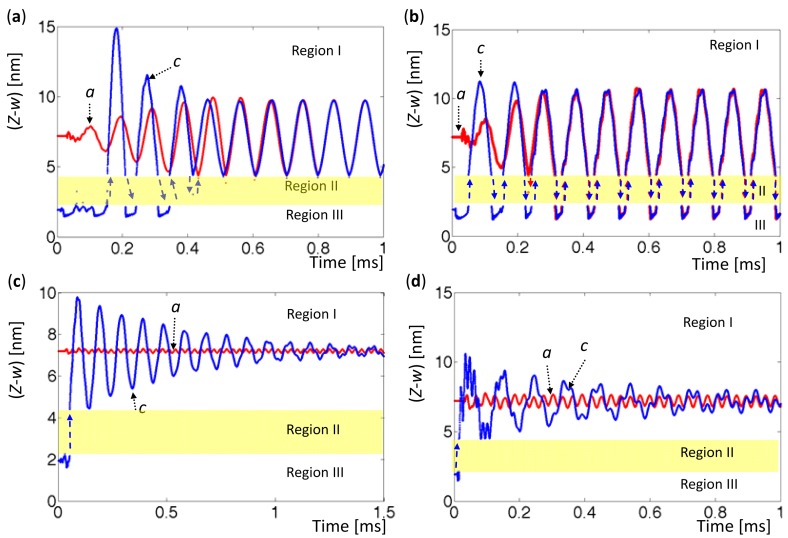
Time histories associated with phase portraits in Figures 8(**c**), 8(**d**), 9(**b**), and 9(**d**). (**a**) *A_m_* = 0.2 nm. (**b**) *A_m_* = 0.4 nm. (**c**) *A_m_* = 0.09 nm. (**d**) *A_m_* = 0.4 nm.

Figure 10(a–d) shows time histories that correspond to the cases in Figures 8(c), 8(d), 9(b), and 9(d), respectively. In Figure 10(a,b), the time histories marked by ‘*a*’ (red) are associated with the oscillation that is driven at initial Point *A* with $\omega_{f}^{A}$, and the time histories marked by ‘*c*’ (blue) are associated with the oscillation that is driven at initial Point *C* with $\omega_{f}^{C}$. All time histories are run from transient states to steady states, and jumps are explicitly indicated. The orbit *c* in Figure 10(a) presents the snapping phenomenon. In Figure 10(b), the orbit *a* and the orbit *c* exhibit hysteresis. Figure 10(c,d) displays the time histories that are associated with the cases in Figure 9(b,d). Clearly, the orbit *c*'s finally converge to the orbit *a*'s, so their phase portraits overlap, as displayed in Figure 9(b,d). These figures also reveal that the time histories have smaller amplitudes and are relatively stable in Region I since they are not driven at their dominate frequency. The tip might jump to the other region, while the driving amplitudes are sufficiently large enough to pass Region II. As a result, the acquired kinetic energy exceeded the superficial potential energy is an important criterion for snapping and intermittency.

### 3.4. Forced Vibration with Various Elevations of AFM Base

**Figure 11.**
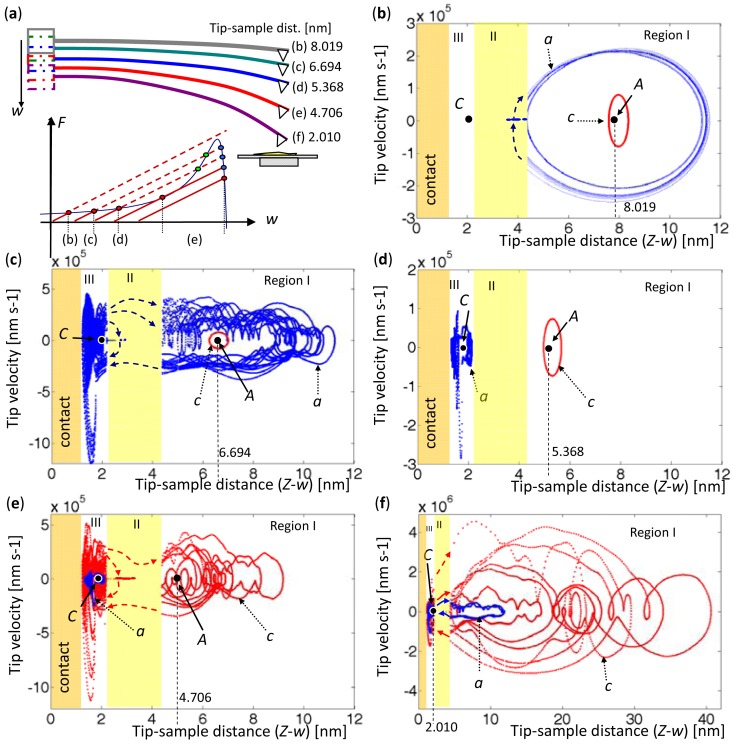
Phase portraits for various tip-sample distances. (**a**) Schematics of microcantilever levels and tip-sample distances. (**b**) *Z-w* = 8.019 nm. (**c**) *Z-w* = 6.694 nm. (**d**) *Z-w* = 5.368 nm. (**e**) *Z-w* = 4.706 nm. (**f**) *Z-w* = 2.010 nm.

With a constant driving amplitude of *A_m_* = 0.4 nm, the forced vibrations were induced at five elevations of AFM base. The associated tip positions were initially in Region I and moved to Region III. Therefore, the tip-sample distances were 8.019, 6.694, 5.368, 4.706, and 2.010 nm, associated with one, three, three, two, and one equilibrium points as presented in Figure 11. The frequencies, $\omega_{f}^{A}=6.5575\times 10^{4}\text{rad}{\text{s}}^{-1}$ and $\omega_{f}^{C}=2.12447\times 10^{5}\text{rad}{\text{s}}^{-1}$, were set. In Figure 11(a–e), the orbits marked by ‘*a*’ (blue) represent the oscillation that is driven at the frequency $\omega_{f}^{A}$, that is the traditional AFM case which drives the microcantilever at dominate frequency. The orbits marked by ‘*c*’ (red) represent that is driven at the frequency $\omega_{f}^{C}$.

In Figure 11(b), the case with a single-equilibrium point involves stable oscillation, and the orbit *a* is amplified when the tip triggers at Point A. In Figure 11(c), the orbit *c* remains stable in Region I, but the orbit *a* that is amplified in Region I and enters Region III, finally becoming chaos. Clearly, AFM can easily detect the signals from the amplitude, and that is the way the traditional AFM used. Moreover, in Figure 11(d), the orbit *a* snaps to oscillate around Point *C* since the oscillation is stable in Region III, and the orbit *c* remains stable in Region I. In Figure 11(e), the tip is initially driven at the equilibrium point *A*, at which is very close to Region II. The orbit *a* finally converges in Region III, and the orbit *c* is amplified because the frequency $\omega_{f}^{C}$ increase the amplitude of oscillation in Region III. In Figure 11(f), the oscillations are driven at the equilibrium point in Region III, and the orbits are amplified to run in three regions and come into contact with the samples. The orbit *c* is distinctly larger than the orbit *a* and it is chaotic, because it is driven at $\omega_{f}^{C}$ and let the microcantilever resonate in Region III.

**Figure 12.**
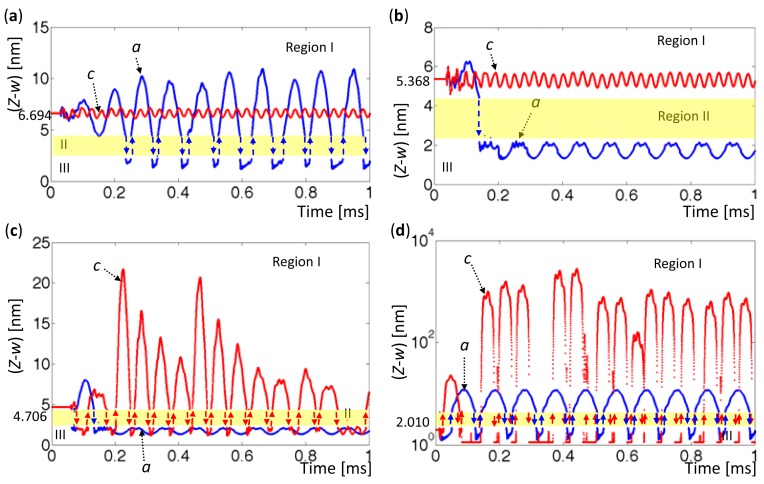
Time histories for various tip-sample distances. (**a**) *Z* − *w* = 6.694 nm. (**b**) *Z* − *w* = 5.368 nm. (**c**) *Z* − *w* = 4.706 nm. (**d**) 2.010 nm.

Figure 12(a–d) display detailed time histories with various tip-sample distances associated with Figure 11(b–e), respectively. The time history marked by ‘*a*’ (blue) represents the oscillation that is driven at $\omega_{f}^{A}$, and the time history marked by ‘*c*’ (red) represents oscillation that is driven at $\omega_{f}^{C}$. In Figure 12(a), intermittency is evident in the time history *a*, which ends in chaos. In Figure 12(b), the time history *a* snaps to Region III, and the time history *c* remains stable in Region I. In Figure 12(c,d), the orbit *c*'s exhibit intermittency and then become chaotic, but the orbit *a*'s remain periodic and exhibit hysteresis.

Comparing Figure 11 with Figure 12 reveals that the oscillations driven at $\omega_{f}^{A}$ exhibit period doubling, snapping, intermittency, and chaos. However, the oscillations driven at $\omega_{f}^{C}$ are stable until the tip touches into Region II. Some early experimental works [15,16] indicated that microcantilever driven at the second eigenvalue improved the sensitivity of an AFM and could avoid chaotic. Indeed, the second eigenvalue was close to the value of the first eigenvalue in Region III, as shown in Figure 5. Therefore, the oscillation driven at $\omega_{f}^{A}$ leads large amplitude and results unstable. However, the oscillation driven at $\omega_{f}^{C}$ has stable amplitudes before the tip comes into Region II, and it provides an obvious amplitude difference after the tip came into Region III. As a result, this specific excitation frequency at $\omega_{f}^{C}$ increased the sensitivity of AFM measurement by enabling the jumps to be recognized and eliminating the uncertainties.

## 4. Conclusions

Numerous nonlinear phenomena occur in AFM experiments, but numerical models have until now failed to be useful in simulating them. Researchers have proposed many methods for avoiding uncertainties in the practical measurements. The tip-jump is one of such nonlinear phenomena, and this investigation proposed a mechanism of jumping. Its initial kinetic energy and the superficial potential energy obtained from the tip-sample distance affect its behavior, which may involve snapping, bistability, hysteresis, intermittency, bifurcation, or chaos.

This investigation noted that characteristics of the microcantilever that varies with the tip-sample distance is a significant requirement in the numerical simulations. This requirement ensures that the eigenvalue of the microcantilever transitions continuously from one associated with the oscillation mode to one associated with the jump mode. Three regions are defined to separate the tip motion range. Region I represents the tip motion before jump, Region II is the region where jump occurred and the first eigenvalue is vanished, and Region III is the interval between jump and contact. This achievement sets the model herein apart from other damped-spring models. This paper reveals that the elevation of the base of AFM can markedly influences the static characteristics of the microcantilever, including its natural frequency and shape functions. The results of forced oscillation show that a jump occurred if and only if the acquired kinetic energy exceeds the superficial potential energy. The results show large excitation amplitudes lead to the acquisition of much kinetic energy. A change in the excitation frequency can increase/reduce its kinetic energy, and to an extent that depends on whether the tip is where the resonant frequency of microcantilever is close to the excitation frequency.

The simulation results reveal that snapping occurs following a jump when the kinetic energy obtained in the region after jump region is too low to enable to jump back. Bistability is occurred by periodic jumps when the kinetic energy obtained in Region I and Region III suffices to maintain periodic jumps. Bistability with contacts may result in hysteresis, and snapping with contacts may lead intermittency or chaos. Moreover, the results indicate that the excitation frequency obtained in Region III can increase the sensitivity of measurement relative to that obtained in Region I. That also can eliminate the uncertainties in AFM detection and can avoid the tip to contacting or destroying the sample.

## Figures and Tables

**Table 1.** Material properties of microcantilever.

Description	Symbol	Si-Si (111) case
Length	*L*	449 μm
Width	*b*	46 μm
Thickness	*h*	1.7 μm
Tip radius	*R*	150 nm
Material density	*ρ*	2,330 kg m^−3^
Static stiffness	*κ*	0.11 N m^−1^
Elastic modulus	*E*	176 GPa
Q factor (air)	*Q*	20
Hamaker (rep.)	*A_1_*	1.3596 × 10^−70^ J m^6^
Hamaker (att.)	*A_2_*	1.865 × 10^−19^ J
